# Ingested Foreign Bodies in the Sigmoid Colon Requiring Earlier Intervention in Patients With Prior Hysterectomy

**DOI:** 10.7759/cureus.61731

**Published:** 2024-06-05

**Authors:** Michael Zaskey, Sarah Williams, Brian Berry, Lou M Smith, Bracken Burns

**Affiliations:** 1 Department of Surgery, East Tennessee State University, Johnson City, USA; 2 Department of Gastroenterology and Hepatology, East Tennessee State University, Johnson City, USA; 3 Department of Surgery, University of Tennessee Medical Center, Knoxville, USA

**Keywords:** hysterectomy, hartmann’s procedure, colonic diverticulosis, partial denture, sigmoid colon, swallowed foreign body

## Abstract

The use of dentures and dental plates is widespread in the adult population. Accidental ingestion of these foreign objects is not uncommon, with the majority of patients having an uneventful passage of the object through the gastrointestinal tract. Of those patients requiring intervention, endoscopy is the most common, followed by surgical removal. We discuss a case of a patient with prior pelvic surgery and diverticulosis causing severe angulation of the bowel, resulting in non-passage of the foreign object requiring surgical intervention.

## Introduction

Foreign body ingestion occurs in the adult population with increased frequency in geriatric patients, and most foreign bodies pass without intervention. The most frequent objects ingested in adults are animal bones, followed by dentures, which are used by 41 million Americans and make up 4-18% of adult foreign body ingestion cases. The proportion of Americans over the age of 65 is expected to increase from 17% to 23% in the next 30 years [1]. Endoscopic removal is required in 20% of cases, while only 1% require surgical intervention [2]. Robust studies evaluating treatments and outcomes are lacking. We report a case of denture ingestion in a 61-year-old female with prior abdominopelvic surgery causing impaction of the dentures requiring operative intervention after failure of conservative therapy, which suggests early endoscopic and operative intervention is warranted in select populations.

## Case presentation

**Figure 1:**
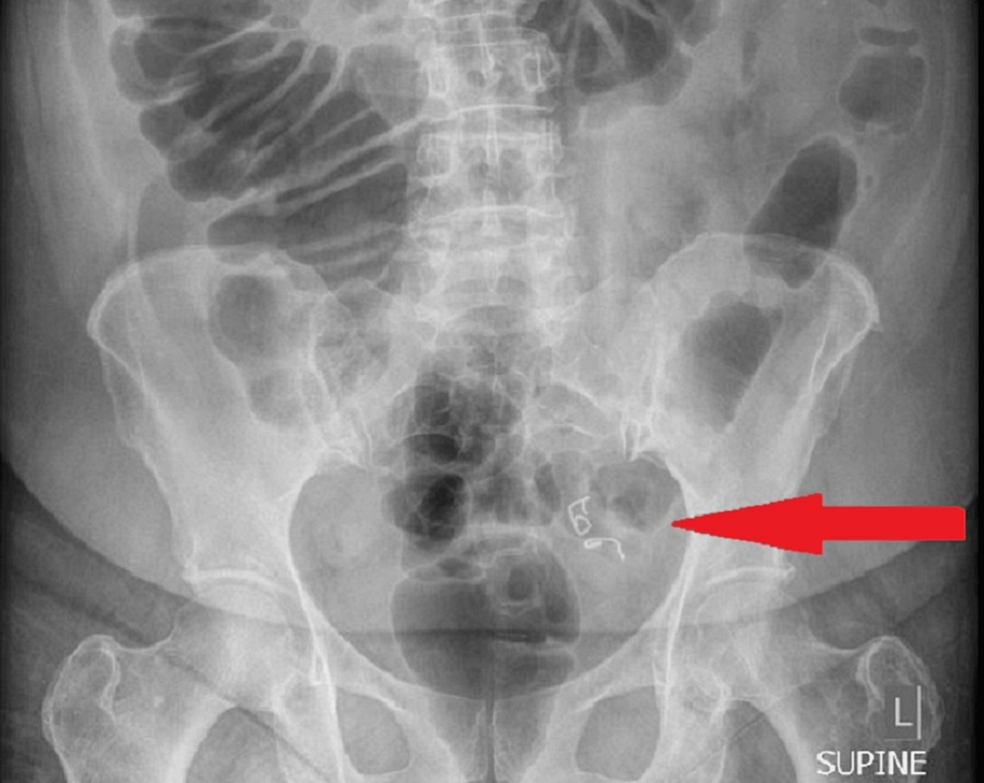
Abdominal X-ray showing the foreign object in the left lower abdomen

**Figure 2:**
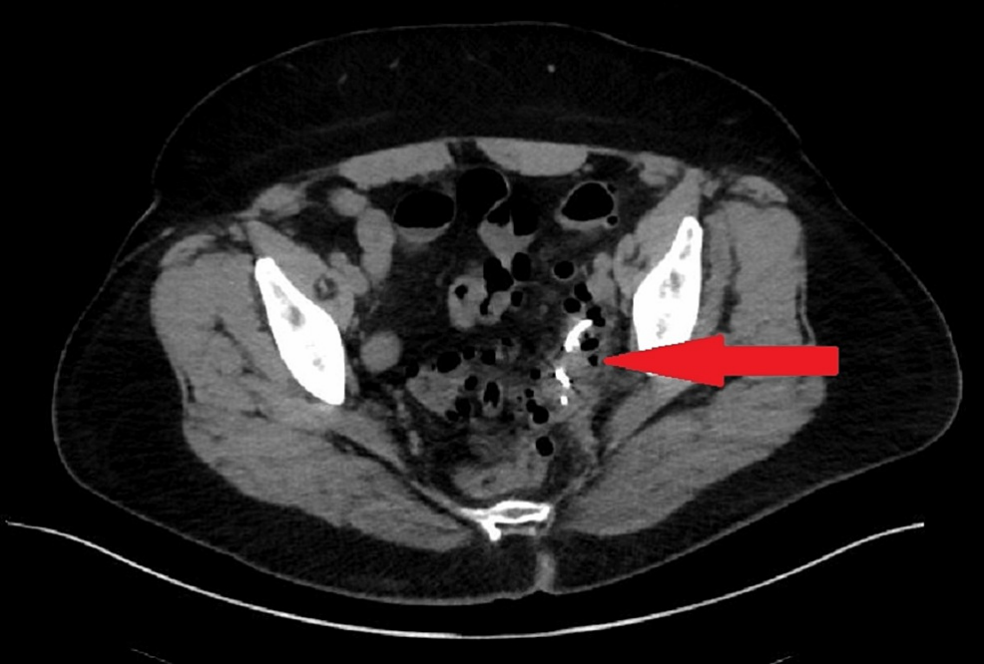
Computed tomography scan with the foreign body in the sigmoid colon with surrounding inflammation

A 61-year-old female presented to the hospital in June 2023 with a chief complaint of generalized abdominal pain. Her past medical history included diverticulosis, chronic obstructive pulmonary disease, daily tobacco use, and fibromyalgia. Her surgical history was significant for a total abdominal hysterectomy, bilateral salpingo-oophorectomy, and bladder suspension. The patient reported swallowing a partial denture plate two weeks prior and presented the following day to a referral hospital, where a computed tomography scan detected a foreign body in the transverse colon. She was discharged home the same day the dentures demonstrated expected advancement through the gastrointestinal tract. On presentation, vital signs were temperature 97.8 Fahrenheit, heart rate 80 beats per minute, blood pressure 119/76, and oxygen saturation 93% on room air. Laboratory work was significant for a white blood cell count of 12,300 uL (3,500 - 10,000/uL). Her physical exam revealed mild tenderness in the lower abdomen without rebound or guarding. A plain abdominal X-ray demonstrated the location of the denture plate (Figure 1). A computed tomography scan showed a foreign body in the sigmoid colon with surrounding inflammatory changes but was without evidence of perforation (Figure 2). The patient was given polyethylene glycol as bowel preparation to assist in the transit of the foreign body as well as in preparation for colonoscopy. Gastroenterology was consulted after three days of bowel stimulation, which failed to promote passage of the partial denture. On colonoscopy, the denture plate was directly visualized 35 to 40 centimeters from the anal verge in a transverse orientation in an area of the rectosigmoid junction that was acutely angled with noted surrounding severe diverticulitis. The foreign body could not be retrieved endoscopically.

An NSQIP score showed a 17.4% risk of complications and a 1.4% risk of death during an exploratory laparotomy. The following day, hospital day 5, the patient was taken to the operating room for direct removal of the foreign body. A lower midline laparotomy was made, and the dentures were palpated in the sigmoid colon. A colotomy was performed with the retrieval of the dentures. The colostomy was closed with a blue-load GIA stapler (Medtronic, Minneapolis, MN, USA) and then oversewn with silk Lembert sutures. After the surgery, the patient progressed well, tolerating a regular diet and returning to bowel function on postoperative day 2. The patient was subsequently discharged from the hospital on postoperative day 3.

The patient returned to the emergency department on postoperative day 9 with severe abdominal pain. Vital signs were a temperature of 97.8 Fahrenheit, a heart rate of 88 beats per minute, a blood pressure of 92/66, and an oxygen saturation of 94% on room air. Laboratory workup revealed a white blood cell count of 10,900 uL (3,500-10,000/uL) and creatinine of 2.04 mg/dL (0.7-1.3 mg/dL). The patient was ill-appearing, with tenderness on abdominal palpation, moderate distention, and a peritoneal finding of rebound tenderness. Computed tomography revealed moderate-volume pneumoperitoneum with free fluid in the pelvis. The patient was taken emergently to the operating room, where a dehiscence of the colotomy closure was noted. A Hartmann’s procedure was performed, and a temporary vacuum-assisted abdominal closure device was placed. The patient was taken to the intensive care unit for further resuscitation. The following day, the patient returned to the operating room for re-exploration, which demonstrated no additional pathology. The abdomen was closed. The patient recovered well and was transferred to the floor to continue her convalescence, where she had returned to bowel function, was tolerating a diet, was ambulatory, and pain was controlled. The patient was scheduled to be discharged to a rehabilitation facility when, on postoperative day 8, the patient developed a sudden onset of altered mental status, diaphoresis, tachycardia, and hypotension. She quickly suffered cardiac arrest, and advanced cardiac life support was performed. The return of spontaneous circulation was achieved, and she was transferred to the intensive care unit for further resuscitation. A discussion with the family regarding the patient’s condition and prognosis was held, and the decision was reached to take only comfort measures for the patient. She expired shortly after.

## Discussion

Our case demonstrates the potential effects of delayed presentation, prior hysterectomy, and diverticulosis on the transit of a sharp-edged foreign body through the gastrointestinal tract. Rutter reports increased angulation of the large bowel, particularly the sigmoid colon, in patients who have undergone surgical removal of a pelvic organ [3]. A 10-year review of 85 patients by Daniels et al. showed that of those who ingested dentures, symptom duration of greater than 24 hours or failure of the object to transit the gastrointestinal tract within four days significantly increased the risk of complications, including abscess, obstruction, and perforation [4]. Ikenberry et al. suggested that surgical removal should be considered for any foreign body that has passed the duodenum but has failed to progress for one week [5]. Lim et al. demonstrated the efficacy of colotomy with primary repair as an acceptable surgical approach in patients with retained intracolonic foreign bodies [6]. A review by Ross et al. looking at retained foreign bodies in patients with known diverticular disease suggested that sigmoid perforation or fistulization requires colectomy but that patients with only inflammation can be managed with less invasive measures [7]. Given the patient's relatively low risk of complications and death per NSQIP calculation and the failure of less invasive treatment modalities, the decision to proceed with colotomy and primary closure was made. The tortuosity, acute angulation of the bowel, and diverticulosis made this a challenging case. In the setting of non-inflamed diverticulosis and previous abdominopelvic surgery, consideration should be made for early resection of the impacted colonic segment with the creation of a colostomy to avoid potentially negative outcomes.

## Conclusions

We recommend the early removal of non-blunt foreign bodies rather than expectant management in patients with a significant delay in presentation and pronounced diverticulosis. In women with prior hysterectomy, surgeons should anticipate difficulty in the passage of foreign bodies of significant size and/or sharp features due to tortuosity, angulation, and narrowing of the sigmoid colon associated with migration into the pelvis and adhesions. A discussion with the patient regarding the creation of an ostomy rather than a colotomy with primary closure should be held, given the potentially devastating complications following a colonic leak.
